# Glycerol Monolaurate to Ameliorate Efficacy of Inactivated Pseudorabies Vaccine

**DOI:** 10.3389/fvets.2022.891157

**Published:** 2022-09-15

**Authors:** Qinghai Ren, Xiaobo Wang, Qingqing Gao, Gaiqin Wang, Xiaochen Chen, Chunxue Liu, Song Gao, Yubao Li

**Affiliations:** ^1^College of Agronomy and Agricultural Engineering, Liaocheng University, Liaocheng, China; ^2^Key Laboratory of Avian Bioproducts Development, Ministry of Agriculture, Yangzhou, China; ^3^Jiangsu Co-innovation Center for Prevention and Control of Important Animal Infectious Diseases and Zoonoses, Yangzhou, China; ^4^Institutes of Agricultural Science and Technology Development, College of Veterinary Medicine, Yangzhou University, Yangzhou, China; ^5^Anyou Biotechnology Group Co., Ltd., Taicang, China

**Keywords:** glycerol monolaurate, pseudorabies virus, inactivated vaccine, immune enhancement, weaned piglets

## Abstract

The present study is aimed to evaluate the effect of glycerol monolaurate (GML) on the growth performance and immune enhancement of pseudorabies virus (PRV)-inactivated vaccine in the early-weaned piglets. One hundred and twenty-five 28-day-old weaned piglets were randomly assigned to a control group (CON, no vaccine and no challenge), challenge control group (C-CON), inactivated PRV vaccine group (IPV), IPV + 500 mg/kg GML group (L-GML), and IPV + 1,000 mg/kg GML group (H-GML) during the entire 28-day experimental period. All the data analyses were performed by one-way analysis of variance (ANOVA) and multiple comparisons. Our results showed that the final weight, average daily gain (ADG), and average daily feed intake (ADFI) of H-GML were the highest in each group, and F/G of H-GML was increased but there was no significant difference with CON (*p* > 0.05). Levels of PRV glycoprotein B (gB) antibody and immunoglobulin in serum of L-GML and H-GML were higher than those of IPV, but only gB antibody levels and immunoglobulin G (IgG) in H-GML were significantly increased (*p* < 0.05). Compared with IPV, the contents of tumor necrosis factor-α (TNF-α), interleukin-6 (IL-6), and interleukin-1β (IL-1β) in serum of L-GML (TNF-α and IL-1β: *p* > 0.05, IL-6: *p* < 0.05, respectively) and H-GML (*p* < 0.01, both) were all decreased, and the content of interleukin-10 (IL-10) in H-GML was increased (*p* > 0.05). Furthermore, reverse transcription-polymerase chain reaction (RT-PCR) experiments proved that L-GML and H-GML were both superior to IPV in inhibiting the expression of TNF-α (*p* < 0.01), IL-6 (*p* > 0.05), and IL-1β (*p* < 0.01) mRNAs and promoting the expression of IL-10 mRNA (L-GML: *p* > 0.05, H-GML: *p* < 0.05, respectively) in the superficial inguinal lymph nodes. Histopathological examination found mild congestion in the lung and inguinal lymph nodes of IPV, while the tissues (brain, lung, and inguinal lymph nodes) of L-GML and H-GML were the same as CON with no obvious lesions. The above results indicate that GML may improve the growth performance of weaned piglets and enhance the immunity of PRV-inactivated vaccine by increasing the levels of PRV gB antibody and immunoglobulin and regulating cytokine levels.

## Introduction

Pseudorabies (PR), also called Aujeszky's disease (AD), is a highly contagious porcine infectious disease caused by pseudorabies virus (PRV), which causes substantial economic losses to the swine agriculture worldwide (1, 2). PRV is a linear double-stranded DNA virus that belongs to the family *Herpesviridae*, subfamily *Alphaherpesvirinae*, and genus *Varicellovirus* (3). Swine are the natural host and reservoir of PRV (4). PRV-infected pigs excreted large amounts of viruses in bodily secretions and excreta (5). Morbidity, mortality, and clinical symptoms of PRV infection in pigs vary with the age (3). For example, neurological signs and high mortality in piglets, respiratory illness, and growth retardation in growing-finishing pigs, as well as abortions or stillbirths in pregnant sows caused by PRV infection (3, 5, 6). At present, the prevention of PRV in China is mainly *via* immunization of gE-deleted live-attenuated vaccines (Bartha-K61 vaccine), which were imported from Hungary in the 1970s (7). Since the widespread application of the vaccine, the morbidity and mortality of newborn piglets in the infected herd have been significantly reduced (8). However, in late 2011, variant PRV strains that appeared in pig farms were vaccinated with Bartha-K61 in China (9, 10). Variant PRV showed strong pathogenicity to pigs of all ages, the Bartha-K61 vaccine did not provide effective protection in the swine industry (7, 11). Studies have shown that the cross-protection ability of the classical live-attenuated vaccines to the variant PRV strains is limited due to the change of antigenicity (7). It is necessary to develop an inactivated vaccine that can effectively resist the variant strain of PRV. Compared with live-attenuated vaccines, inactivated vaccines are safer but cannot induce cellular immune responses, and cellular immunity played a crucial role in PRV protective immunity (12). Therefore, it is of great clinical significance to study methods to improve the immune effect of inactivated vaccines and enhance the disease resistance of swine.

Glycerol monolaurate (GML), which is the monoglyceride derivative of lauric acid that is generally considered to be more biologically potent than medium-chain fatty acid (MCFA) *in vitro*, naturally existed in the breast milk and coconut oil (13, 14). It not only has excellent emulsification properties but also has a strong ability to inhibit the growth and proliferation of gram-positive bacteria and enveloped viruses *in vitro*, as well as the production of bacterial virulence factors (15–17). The results of Thomas et al. showed that adding GML and 1.0% 1:1:1 MCFA had the same effect, which could significantly improve the average daily gain (ADG), average daily feed intake (ADFI), and gain to feed ratio (G:F) of nursery pigs, when compared with pigs fed a control diet (18). Ghalib found that 4 g/kg of GML significantly increases body weight gain (BWG) and food conversion ratio, improvement of pro-inflammatory cytokines (tumor necrosis factor-α [TNF-α] and interleukin [IL]-12), and total antioxidant capacity (TAC) (19). Liu et al. demonstrated that dietary GML improved performance, intestinal morphology, and muscle amino acids in broilers *via* manipulating community, function, and metabolites of gut microbiota (20).

Currently, there were a few studies on GML as an immune enhancer to improve the effect of inactivated vaccines. The purpose of this experiment was to study the enhancement effect of oral GML in weaned piglets on the immunity of PRV-inactivated vaccine and provide a reference for using MCFAs and derivatives to improve the immune effect of inactivated vaccine in the clinic.

## Materials and Methods

### Ethics Statement

All animal procedures related to experiment were carried out in strict accordance with the animal care and ethical standards, approved by the ethics committee of Yangzhou University (Jiangsu province, China). All efforts were made to reduce the suffering of animal.

### Animal Experiment Design

One hundred and twenty-five weaned piglets (D × L × Y, 28-day old) were randomly distributed to 5 treatment groups: the control group (CON, no vaccine and no challenge), challenge control group (C-CON), inactivated PRV vaccine group (IPV), IPV + 500 mg/kg GML group (L-GML), and IPV + 1,000 mg/kg GML group (H-GML). Each group consisted of 5 replications per group, 5 piglets per replication. Entire experimental period lasted for 28 days, feed and water were available *ad libitum*. All piglets were adapted to the environment for 1 week. Except for CON and C-CON, the other groups were immunized with PRV-inactivated vaccine on the 7 day of the experiment. On the 14th day, a challenge test was conducted (except CON), and the challenge dose was 1 × 10^6^ Median Tissue Culture Infectious Dose (TCID_50_; vPRV/XJ5 strain from our laboratory). The animal experiment design of this article is presented in Table 1. The basic diet was prepared according to the National Research Council (21) without antibiotics or medicine. The feeding conditions, management, and sanitary environment of all experimental piglets are the same. Daily temperature measurement and clinical symptom observation were performed after the challenge. On the 28th day of the test, blood was collected from the anterior vena cava. After clotting, the blood was centrifuged at 3,500 r/min for 20 min to separate the serum. At the same time, the piglets were dissected, the corresponding tissues were taken out, and stored at −80°C together with the serum until use.

**Table 1:** Animal experimental design.

**Group**	**Diet**	**Vaccine**	**Challenge**
CON	basic diet	unvaccinated	unchallenged
C-CON	basic diet	unvaccinated	challenged vPRV/ XJ5 strain (day 14)
IPV	basic diet	PRV inactivated vaccine (day 7)	challenged vPRV/ XJ5 strain (day 14)
L-GML	basic diet+ 500 mg/kg GML	PRV inactivated vaccine (day 7)	challenged vPRV/ XJ5 strain (day 14)
H-GML	basic diet+ 1000 mg/kg GML	PRV inactivated vaccine (day 7)	challenged vPRV/ XJ5 strain (day 14)

### Measurement on Growth Performance

The piglets were weighed at the beginning and end of the experiment and fasted for 8 h before weighing. BWG and feed intake (FI) were recorded weekly for the piglets in each group. Calculated the ADG according to the initial weight and final weight of the piglets, calculated the ADFI according to the feed consumption, and finally the ratio of feeds to weight (F/G) was calculated.

### Blood Analysis

According to the manufacturer's instructions of PRV glycoprotein B (gB) antibody detection kit (IEDXX, USA), the level of specific PRV gB antibody in serum was detected by blocking enzyme linked immunosorbent assay (ELISA). Blocking rate of PRV gB antibody = [1 – S (value of sample *D*_450nm_)/N (value of negative control *D*_450nm_)] × 100%. The serum levels of immunoglobulin [immunoglobulin G (IgG), immunoglobulin A (IgA), and immunoglobulin M (IgM)] were determined using ELISA kits that were purchased from Shanghai Fusheng Industrial Co., Ltd. (Shanghai, China). Serum cytokines (TNF-α, IL-6, IL-1β, and IL-10) were measured using the double antibody sandwich method of ELISA, according to the instructions (Abcam, United Kingdom).

### Fluorescence Quantitative RT-PCR Analysis

According to the manufacturer's instructions, the total RNA in the inguinal lymph nodes was extracted using RNAprep Pure Tissue Kit (Tiangen Biotech Co., Ltd., China). Then, 1 μg of RNA was reverse transcribed into cDNA from total RNA using a PrimeScript^TM^ RT Master Mix (Perfect Real Time) Kit (Takara Biomedical Technology Co., Ltd., China). The primer sequences for TNF-α, IL-6, IL-1β, IL-10, and glyceraldehyde-3-phosphate dehydrogenase (GAPDH) are represented in Table 2. Reactions of qRT-PCR were run with the AceQ^®^ qPCR SYBR^®^ Green Master Mix Kit (Vazyme Biotech Co., Ltd., China), according to the instructions of the manufacturer. The PCR amplification parameters were 95°C for 5 min followed by 40 cycles of 95°C for 10 s and 60°C for 30 s. GAPDH gene was served as the internal reference to normalize target gene expression levels. The relative fold-change in the expression of target genes was calculated by the 2–ΔΔCt (Ct: cycle threshold) method. The abundance of mRNA transcripts per sample was evaluated three times.

**Table 2:** Primer sequences used for the reverse transcription-polymerase chain reaction (RT-PCR) analysis.

**Primer name**	**Sequence (5^**′**^-3^**′**^)**	**Product size (bp)**	**Genbank accession number**
TNF-α	F: AGACACCATGAGCACTGAGAGCAT R: GAAGTGCAGTAGGCAGAAGAGCGT	166	X 57321.1
IL-6	F: CAGGAACGAAAGAGAGCTCCATCT R: TCACCTTTGGCATCTTCTTCCAG	159	NM 214399.1
IL-1β	F: CTACCCTCTCCAGCCAGTCTTCATT R: GCCATCAGCCTCAAATAACAGGTC	130	NM 214055.1
IL-10	F: GAAGCTCACAACTCCGAAGGAATC R: ATCGAGGGAGCCAGAATTGGTC	132	JQ 687536.1
GAPDH	F: TCAAGATCGTCAGCAATGCCTC R: GGTAGAAGCAGGGATGATGTTCTG	203	NM 001206359.1

### Histopathological Analysis

Tissues of brain, lung, and superficial inguinal lymph nodes were fixed in 4% paraformaldehyde for 24 h and embedded in paraffin (22). The paraffin section was cut into slices of 5 μm, stained with hematoxylin and eosin (H&E). Histopathological examination of the piglet tissues was performed using light microscope (OLYMPUS, Japan).

### Statistical Analysis

All data are expressed as mean ± standard deviation (mean ± SD). The data were analyzed by one-way analysis of variance (one-way ANOVA), followed by least significant difference (LSD) multiple comparison tests with SPSS version 21.0 (SPSS Inc., Chicago, IL, USA) (23). Difference between samples was considered to be significant when *p* < 0.05. GraphPad Prism 8 (GraphPad Software Inc. San Diego, CA, USA) was used for statistical analysis (22).

## Results

### Growth Performance

As shown in Table 3, there is no significant difference in the initial weight of each group (*p* > 0.05). In C-CON, IPV, and L-GML, the final weight was lower than CON, but only C-CON had a significant difference (*p* < 0.05). The final weight of H-GML was the highest in each group, there was no significant difference with CON (*p* > 0.05). In terms of weight gain, both H-GML and L-GML were significantly higher than C-CON and IPV (*p* < 0.05), and HGML was even higher than CON, but the difference was not significant (*p* > 0.05). Compared with CON, ADG and ADFI in C-CON and IPV were decreased significantly (*p* < 0.05). In addition, ADG and ADFI of L-GML were reduced but the differences were not significant (*p* > 0.05), while H-GML was increased instead (*p* > 0.05). An increase in F/G was found (*p* < 0.05) in C-CON when compared with CON piglets, whereas there was no significant effect on the F/G of piglets from IPV, L-GML, and H-GML (*p* > 0.05).

**Table 3:** The effects of different treatments on growth performance of weaned piglets.

**Item**	**CON**	**C-CON**	**IPV**	**L-GML**	**H-GML**
Initial weight (kg)	7.65 ± 0.37	7.73 ± 0.40	7.82 ± 0.44	7.67 ± 0.42	7.62 ± 0.39
Final weight (kg)	21.69 ± 3.09^a^	18.91 ± 2.44^b^	19.81 ± 2.90^ab^	20.59 ± 3.63^ab^	22.07 ± 3.21^a^
Weight gain (kg)	14.04 ± 2.08^a^	11.18 ± 2.46^b^	11.99 ± 2.93^b^	12.92 ± 3.77^ab^	14.45 ± 3.18^a^
ADG (kg)	0.50 ± 0.11^a^	0.40 ± 0.09^b^	0.43 ± 0.10^b^	0.46 ± 0.13^ab^	0.52 ± 0.11^a^
ADFI (kg)	0.88 ± 0.22^a^	0.78 ± 0.18^b^	0.80 ± 0.19^b^	0.84 ± 0.23^ab^	0.92 ± 0.23^ab^
F/G	1.76 ± 0.11^a^	1.94 ± 0.12^b^	1.88 ± 0.10^ab^	1.83 ± 0.12^ab^	1.78 ± 0.13^a^

Values with different letter superscripts mean significant difference (*p* < 0.05). No letter or the same letter superscripts represent no significant difference (*p* > 0.05).

### PRV gB Antibody Levels

**Figure 1:**
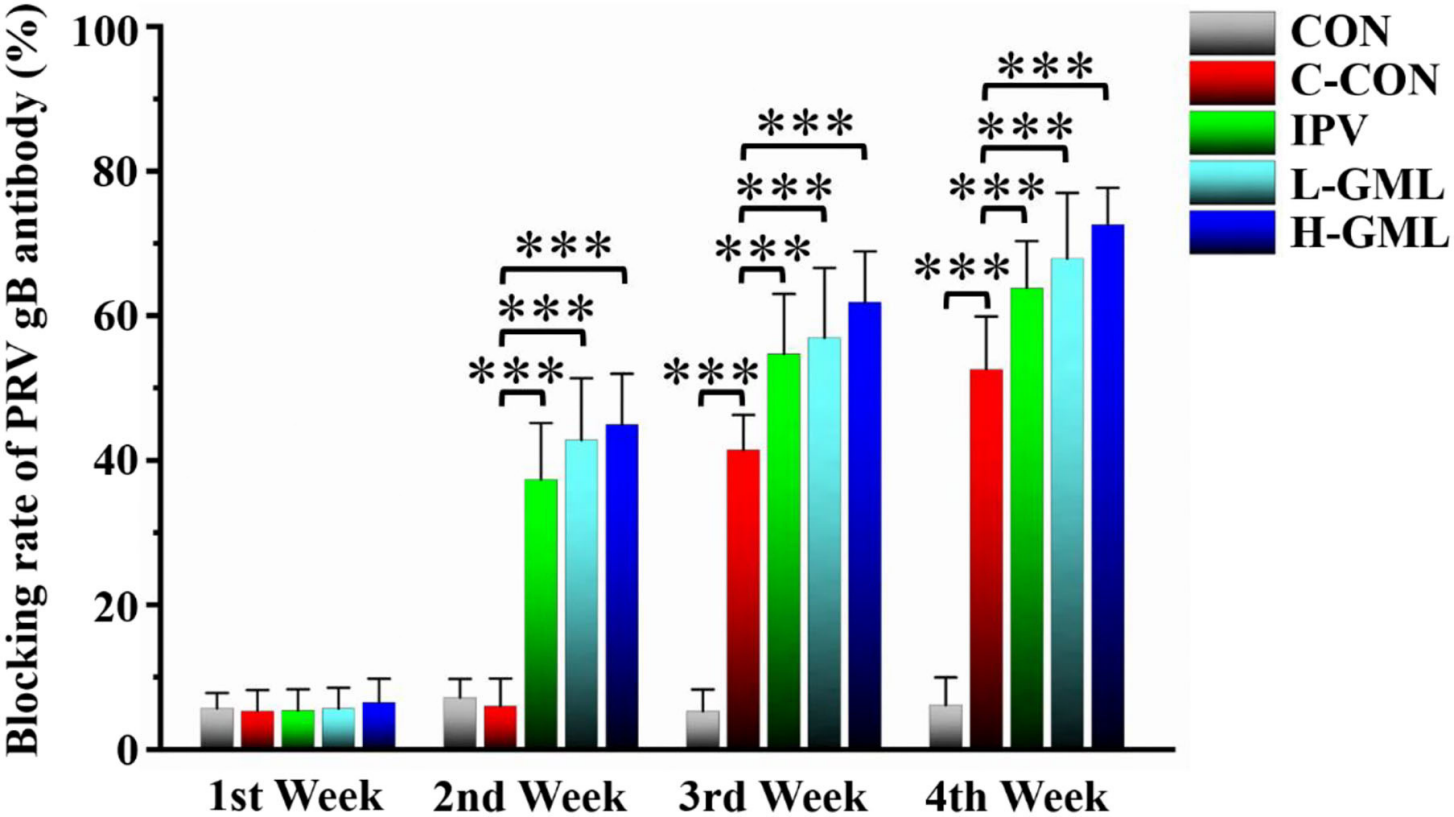
Pseudorabies virus glycoprotein B (PRV gB) antibody levels in serum of experimental pigs. The results are presented as mean ± SD. Dissimilar numbers of stars represent significant differences (*: *p* < 0.05, **: *p* < 0.01, ***: *p* < 0.001).

Figure 1 shows that there is no significant difference in PRV gB antibody levels of each group at the first week (*p* > 0.05). Compared with CON, the levels of PRV gB antibody in IPV, L-GML, and H-GML were extremely significantly higher than those in C-CON (*p* < 0.001) from the second week to the end of the experiment. Moreover, the antibody levels of L-GML and H-GML were both higher than IPV, but only H-GML had a significant difference (*p* < 0.05).

### Serum Levels of Immunoglobulin

**Figure 2:**
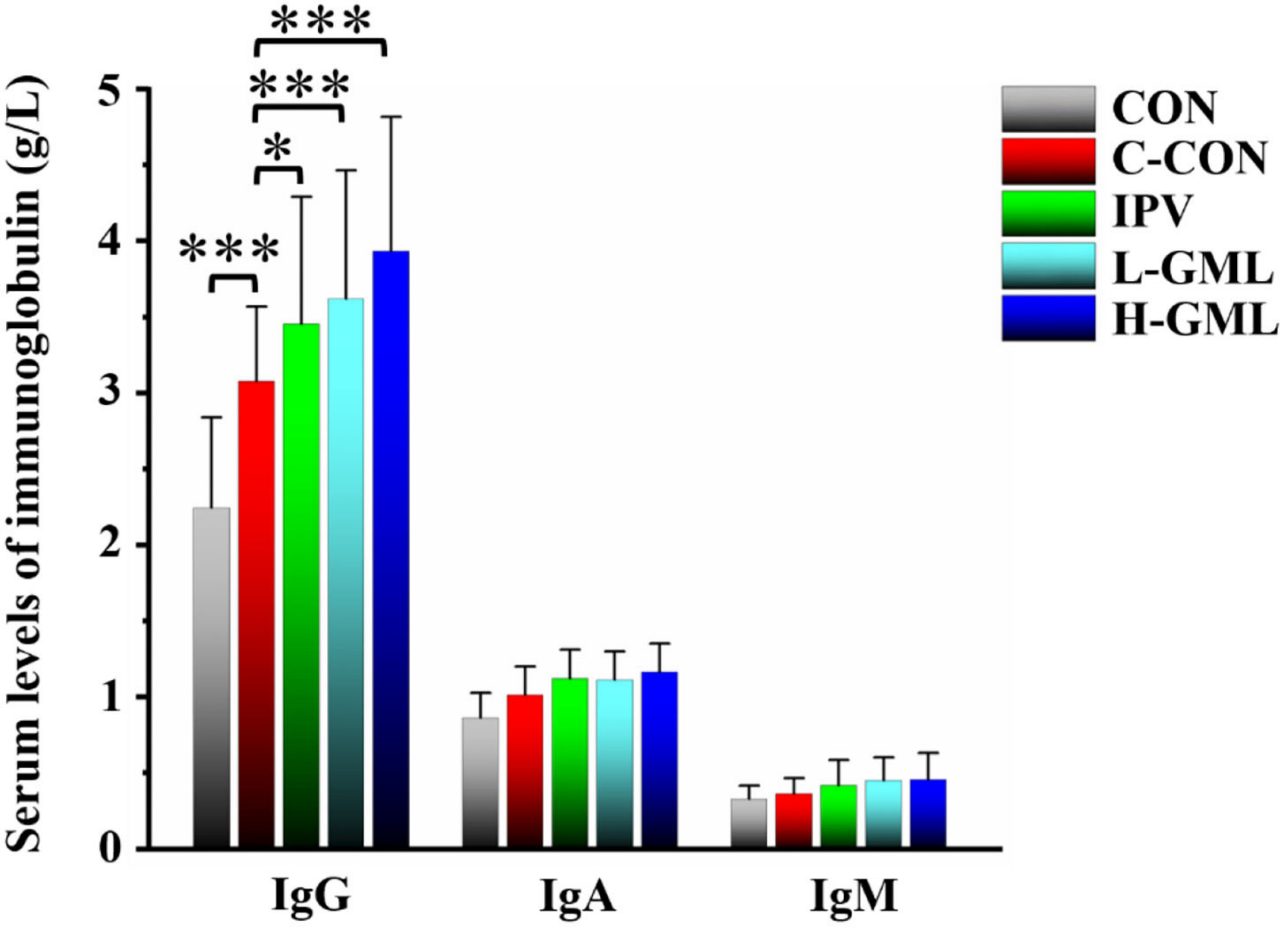
Levels of immunoglobulin G (IgG), immunoglobulin G (IgA), and immunoglobulin G (IgM) in serum of experimental pigs. The results are presented as mean ± SD. Dissimilar numbers of stars represent significant differences (*: *p* < 0.05, **: *p* < 0.01, ***: *p* < 0.001).

As shown in Figure 2, the serum IgG content of C-CON is extremely significantly higher than CON (*p* < 0.001). Except that the serum IgG content of IPV was significantly higher than C-CON (*p* < 0.05), both L-GML and H-GML were extremely significantly higher than C-CON (*p* < 0.001). Although the levels of IgA and IgM in the serum of IPV, L-GML, and H-GML were higher than those of C-CON, there was no obvious difference among the groups (*p* > 0.05).

### Serum Levels of Cytokines

**Figure 3:**
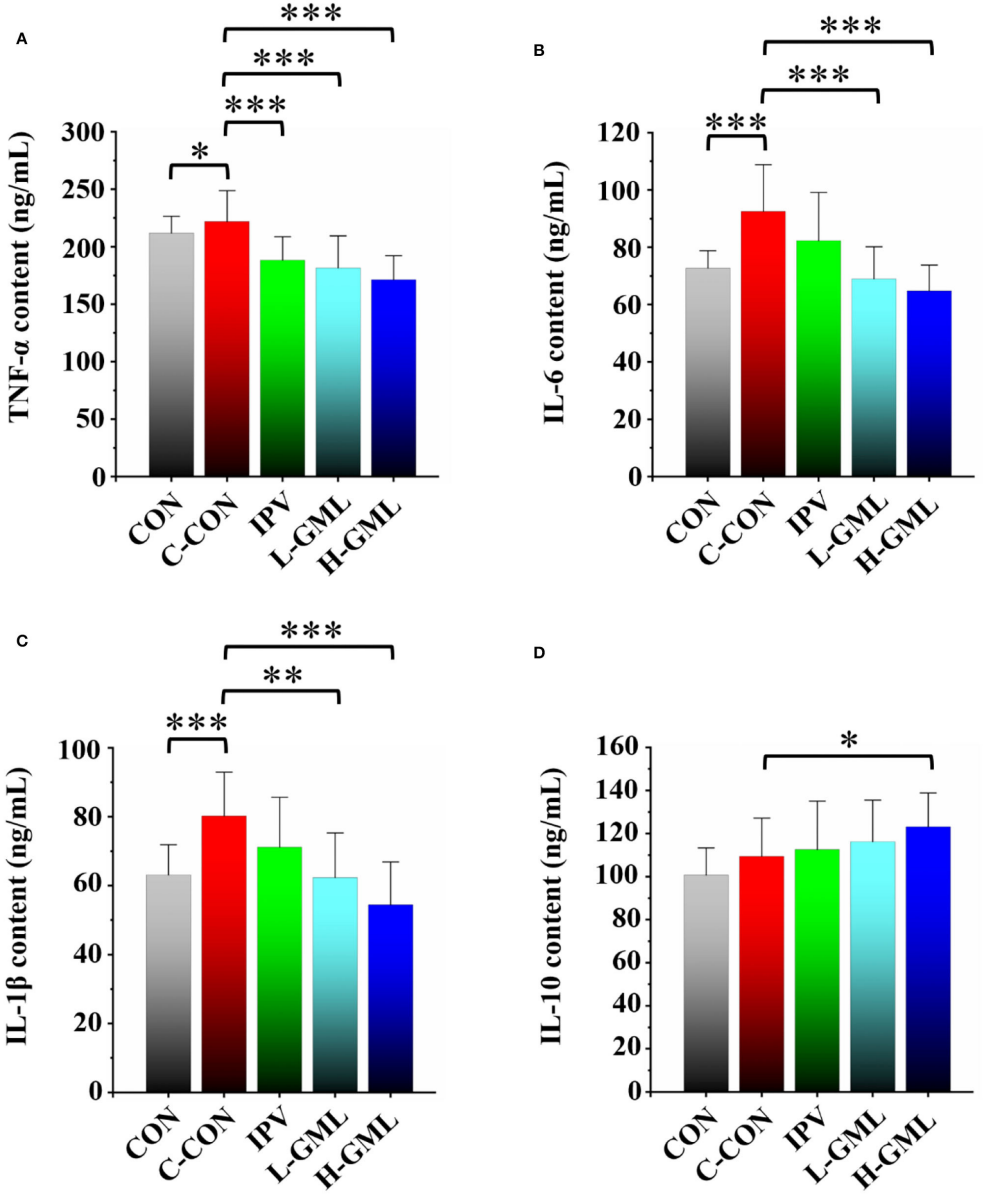
Levels of cytokines **(A)** TNF-α, **(B)** IL-6, **(C)** IL-1β, and **(D)** IL-10 in serum of experimental pigs. Dissimilar numbers of stars represent significant differences (*: *p* < 0.05, **: *p* < 0.01, ***: *p* < 0.001).

The results showed that TNF-α level in serum of the piglets from CON was significantly reduced (*p* < 0.05) and the other groups were extremely significantly decreased (*p* < 0.001), when compared with the C-CON (Figure 3A). At the levels of IL-6 and IL-1β, no differences between IPV and C-CON were observed (*p* > 0.05) but the other groups were extremely significantly lower than C-CON (*p* < 0.01, Figures 3B,C). Except for IL-10 level of H-GML was significantly higher than that of C-CON (*p* < 0.05), there was no difference in the other groups (*p* > 0.05, Figure 3D).

#### Gene Expression of Cytokines

**Figure 4:**
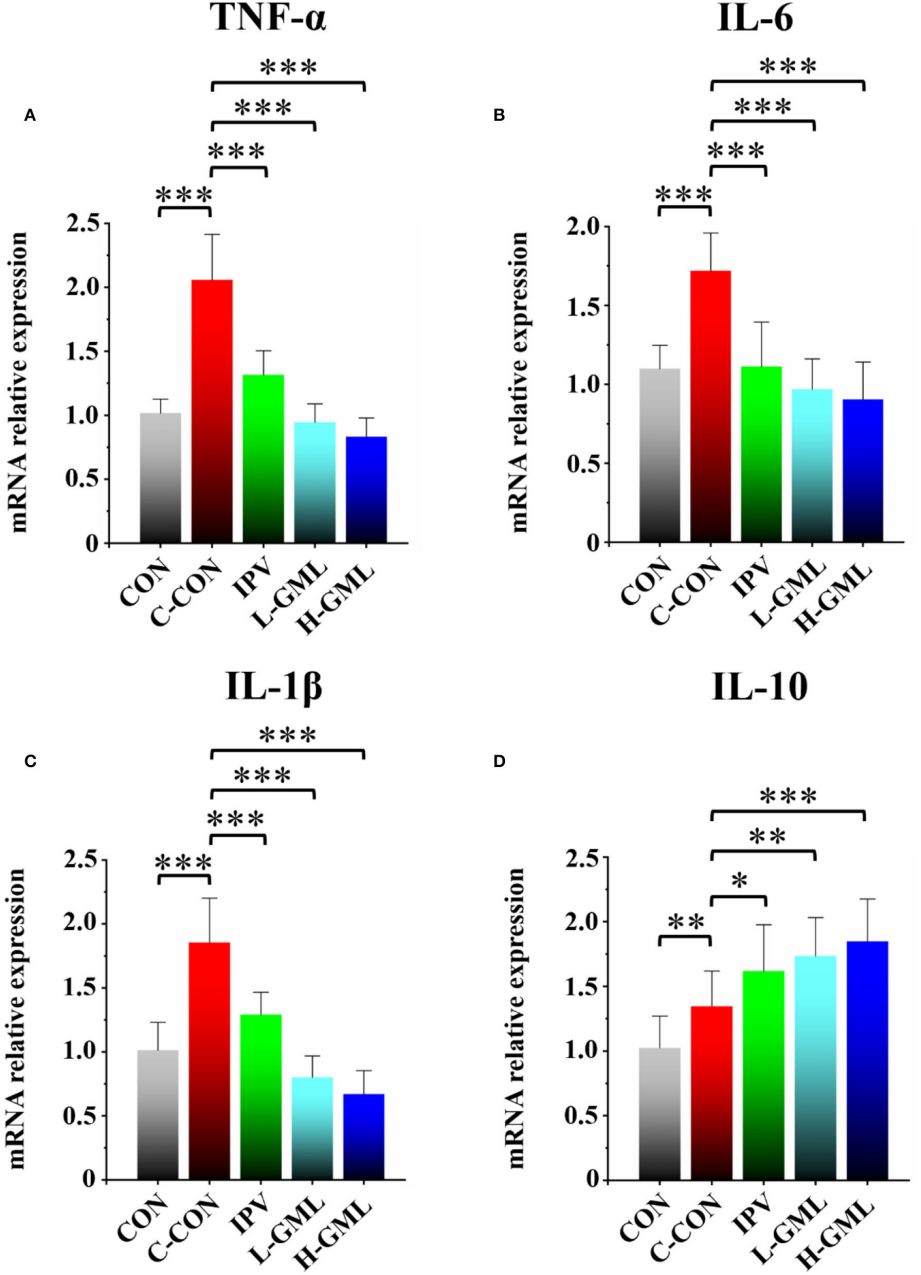
Cytokine **(A)** TNF-α, **(B)** IL-6, **(C)** IL-1β, and **(D)** IL-10 level of the superficial inguinal lymph nodes of experimental pigs. Dissimilar numbers of stars represent significant differences (*: *p* < 0.05, **: *p* < 0.01, ***: *p* < 0.001).

As presented in Figure 4, piglets from C-CON have obvious significantly increased TNF-α, IL-6, and IL-1β mRNAs in the superficial inguinal lymph nodes (*p* < 0.001), when compared with other groups. Although there was no statistical significant in the mRNA expression level of IL-6 (*p* > 0.05), the mRNAs of TNF-α and IL-1β in L-GML and H-GML were extremely significantly higher than those of IPV (*p* < 0.001, Figures 4A–C). Compared with C-CON, IL-10 mRNAs in IPV, L-GML, and H-GML were increased, and the differences were all significant (*p* < 0.05), but a significant decrease in CON was found (*p* < 0.01, Figure 4D).

### Histopathological Analysis

**Figure 5:**
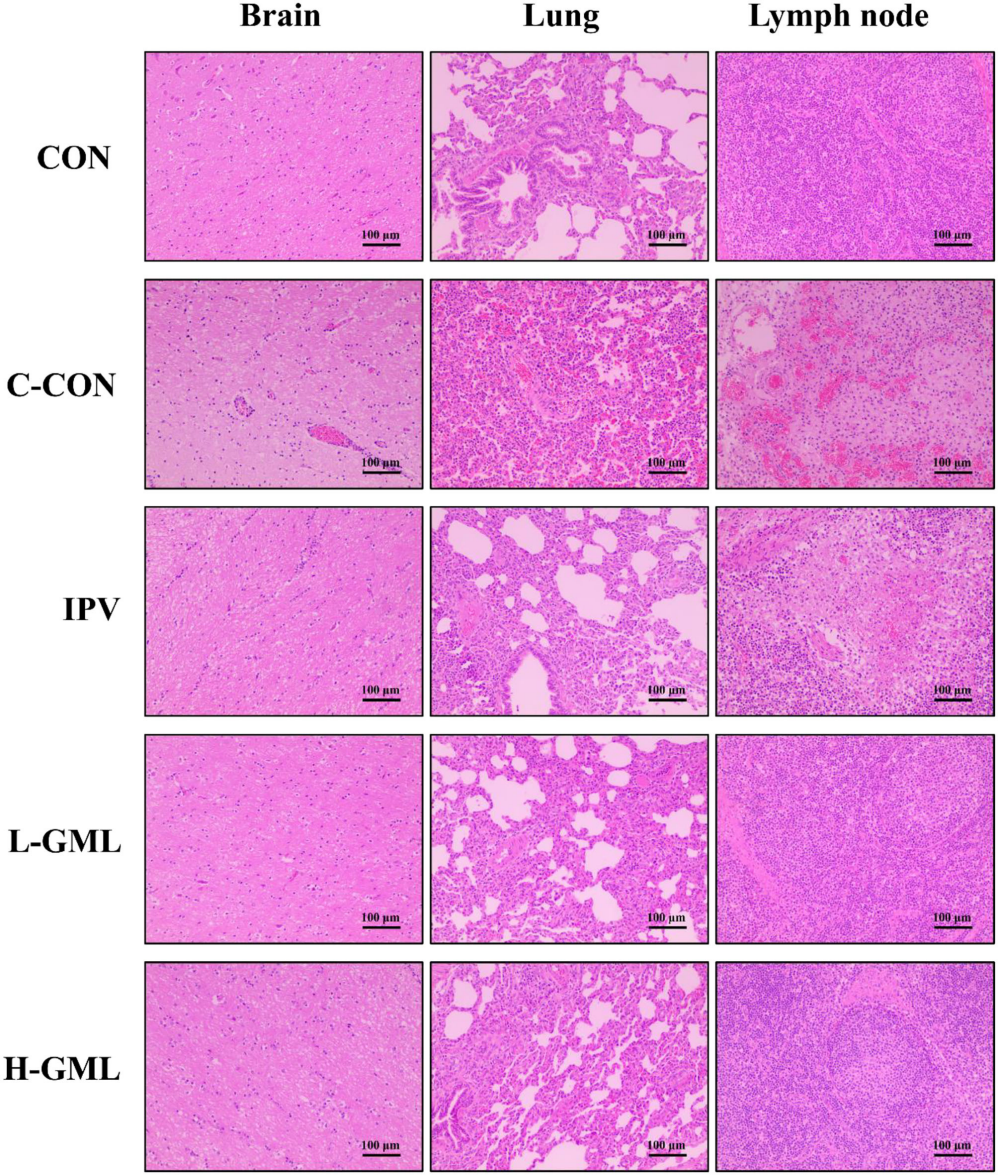
Histopathological observations in the brain, lung, and superficial inguinal lymph nodes of experimental pigs.

As shown in Figure 5, the brain is congested, and there is mild-to-moderate lymphocyte infiltration around the blood vessels, forming a lymphocytic vascular cuff in C-CON. In addition, the pulmonary veins from the tissues of C-CON were congested extensively, and there was bleeding into the inguinal lymph nodes. In IPV, no lesions were observed in the brain, but there was mild congestion in the lungs and inguinal lymph nodes, and a few lymphocytes in the lymph nodes were necrotic. The tissues of CON, L-GML, and H-GML showed no obvious pathological changes.

## Discussion

Pseudorabies virus is prevented and controlled by the use of vaccination with attenuated live and inactivated vaccines (24). Attenuated live vaccine played a key role in the attempt to control or even eradicate PR (25–28). However, safety is one of the most important indicators for evaluating qualified vaccines. Kong et al. found that attenuated live vaccine (Bartha-K16) caused an outbreak of PR in sheep (29). Attenuated live vaccine, because of incomplete attenuation and reversion to pathogenic virus, would lead to disease outbreaks in severe cases (30, 31). Unfortunately, severe PR outbreaks took place in pigs vaccinated with Bartha-K61 vaccine in China, indicating that the PRV variant has proved to be more virulent (32–35). Therefore, inactivated vaccines were widely used in China, which can generate strong and durable specific antibodies in serum, and were safer than attenuated live vaccines (36, 37). However, inactivated vaccine had little ability to induce a cellular immune response in animals (38). This results in live vaccines often being more effective than inactivated preparations, especially when they contain high viral titers and are adjuvant (39). Thus, it is necessary to develop new methods to enhance the efficacy of inactivated vaccines against PRV, and it has become a trend of current research.

### Combination of GML and Inactivated Vaccine Could Improve the Reduction of Piglet Growth Performance Caused by PRV

Recently, it has been reported that using inactivated vaccines, supplemented with adjuvants, can effectively enhance the immune effect of vaccines (38, 40). Yoo et al. found that muramyl dipeptide derivatives enhance immunogenicity of a hantavirus-inactivated vaccine (41). Rivera et al. reported that adding ginseng to the inactivated antigen of porcine parvovirus (PPV) could significantly increase amount of cytokines, specific antibody titers, and elicited a balanced Th1 and Th2 immune response in the serum of Balb/c mice (42). Sun et al. evaluated the immune-enhancing activity of polysaccharides from the Rhizoma of Atractylodis Macrocephalae Koidz (RAMPS), and the results showed that RAMPS could significantly enhance T lymphocyte proliferation (43). GML is not only an excellent food emulsifier but also a safe, efficient, and broad-spectrum bacteriostatic agent and has excellent antiviral function, which makes it a potential vaccine immune enhancer (17, 44–46). Therefore, the purpose of this experiment was to evaluate the immune-enhancing function of oral GML in weaned piglets on PRV-inactivated vaccine.

Studies have shown that MCFAs are more easily absorbed by newborn piglets than long-chain fatty acids (LCFAs) (47). MCFAs could directly supply energy for piglets and affect the composition of the gut microbiota through antibacterial properties, thereby improving the survival rate of weaned piglets and improving growth performance (48, 49). As a representative of MCFAs, GML could not only significantly improve the ADG, ADFI, and G:F of weaned piglets but could also increase the weight gain and food conversion ratio in chicks (18, 19). Our results showed that ADG of IPV (*p* > 0.05), L-GML (*p* > 0.05), and H-GML (*p* < 0.001) was increased and F/G was decreased (IPV and L-GML: *p* > 0.05, H-GML *p* < 0.001, respectively), when compared with C-CON. Furthermore, the ADG of H-GML was the highest in each group. Although the F/G of H-GML was higher than that of CON, there was no significant difference (*p* > 0.05). Therefore, the above results showed that the combination of GML- and PRV-inactivated vaccine could significantly improve the reduction of piglet growth performance caused by PRV when compared with the single use of the vaccine.

### GML Could Increase PRV gB Antibody Levels, the Immunoglobulin Content in Serum, and Regulate Cytokines in Serum and Lymph Nodes

Central to the membrane fusion event is gB, which is the most conserved envelope protein across the herpesvirus family (50). Previous studies showed that PRV gB was a major target of host immune defense due to its involvement in virus infection of cells (51, 52). Detection of PRV gB antibody levels in serum by ELISA could evaluate the overall immunity level of pigs and then guide vaccine immunization. In this study, we found that GML could enhance the immune effect of inactivated vaccines by increasing PRV gB antibody levels in serum. Ig is one of the major anti-infective components in blood, colostrum, and breast milk, protecting the body from harmful bacteria, viruses, and other environmental pathogens by either binding to them or forming an encapsulating barrier (53). Gomez et al. found that feeding porcine immunoglobulin could improve growth performance and survival in colostrum-deficient piglets (54). It could be seen that immunoglobulins were of great significance to enhance the immune ability and resistance to infection of piglets (55). Our results showed that levels of immunoglobulins in serum of L-GML and H-GML were higher than those of IPV, especially the levels of IgG in H-GML were significantly increased (*p* < 0.05). This study indicated that GML might assist the vaccine by increasing the immunoglobulin content in piglets to enhance humoral immunity, thereby improve the ability of piglets to resist PRV infection.

Cytokines as immunomodulatory proteins are secreted by a variety of cells, especially those involved in immune response, such as macrophages and T cells (56, 57). Researchers found that co-administrating of recombinant porcine interleukin-2 (IL-2) could enhance protective immune responses to PRV-inactivated vaccine in pigs (12). GML could modulate T-cell proliferation by affecting the cell signal cascade leading to the regulation of IL-2 production (15). In this study, our results showed that the addition of GML reduced the expression of pro-inflammatory cytokines (TNF-α, IL-6, and IL-1β) and increased the secretion of anti-inflammatory cytokine (IL-10) in piglet serum, when compared with the single use of inactivated vaccine. The lymphatic system plays an important role in host immune defense and body metabolism [57, 58]. Therefore, we collected the superficial inguinal lymph nodes of piglets to detect the differences in mRNA expression of cytokines. Our RT-PCR experiments demonstrated that the differences of cytokine mRNA expression in lymph nodes were similar to those of serum cytokines. The results suggested that GML could selectively regulate cytokine expression in serum and lymph nodes to improve the cellular immune function of piglets and the immune effect of the PRV-inactivated vaccine.

### GML Could Cooperate With an Inactivated Vaccine to Prevent and Improve Histopathological Changes Caused by PRV

According to the previous research in our laboratory, the pathological lesions in challenge only pigs included dilated and congested blood vessels in the brain, a large number of eosinophils infiltrating the superficial inguinal lymph nodes and edema, congestion, hemorrhaging, and massive neutrophil infiltration in the alveolar space (22). Thus, in order to observe histopathological changes in piglet tissues under different treatment conditions, we performed pathological analysis of brain, lung, and superficial inguinal lymph nodes. Our results of this study observed that mild congestion in the lungs and inguinal lymph nodes of IPV and a small number of lymphocytes in the lymph nodes were necrotic. The tissues (brain, lung, and inguinal lymph nodes) of GML adding group were the same as CON with no obvious lesions. This indicated that GML could cooperate with inactivated vaccine to prevent and improve histopathological changes caused by PRV.

## Conclusion

Our study demonstrated that GML might improve growth performance of weaned piglets and enhance the immunity of PRV-inactivated vaccine by increasing the levels of PRV gB antibody and immunoglobulin, as well as selectively regulating cytokine levels. The above findings laid a solid foundation for future studies on the effect of MCFAs and derivatives to improve the immune effect of PRV-inactivated vaccine in the clinics.

## Data Availability Statement

The original contributions presented in the study are included in the article/supplementary material, further inquiries can be directed to the corresponding author.

## Ethics Statement

The animal study was reviewed and approved by Yangzhou University (Jiangsu Province, China). Written informed consent was obtained from the owners for the participation of their animals in this study.

## Author Contributions

QR designed experiments and wrote the manuscript. XW, QG, GW, XC, CL, SG, and YL were involved in discussing data and modifying the manuscript. All authors read and agreed to the published manuscript.

## Funding

This study was funded by grants from the Key R&D Program of Jiangsu Province (BE2020320), the National Key R&D Program (2016YFD0500704-2), the Novel Agricultural Research Program of Jiangsu Province (SXGC [2017] 231), funding from the Priority Academic Program Development of Jiangsu Higher Education Institutions (PAPD) and Science and Technology Support Program of Jiangsu Province (BE2014355), and the earmarked fund for Jiangsu Agricultural Industry Technology System (JATS [2018]221).

## Conflict of Interest

Authors GW, XC, and CL were employed by Anyou Biotechnology Group Co., Ltd. The remaining authors declare that the research was conducted in the absence of any commercial or financial relationships that could be construed as a potential conflict of interest.

## Publisher's Note

All claims expressed in this article are solely those of the authors and do not necessarily represent those of their affiliated organizations, or those of the publisher, the editors and the reviewers. Any product that may be evaluated in this article, or claim that may be made by its manufacturer, is not guaranteed or endorsed by the publisher.
